# Comprehensive pan-effectome investigation reveals central effector genes in woody plant pathogen Botryosphaeriaceae

**DOI:** 10.1128/aem.01619-25

**Published:** 2026-04-06

**Authors:** Aining Guo, Qikai Xing, Hanyin Zhang, Ishara S. Manawasinghe, Wei Zhang, Xuncheng Wang, Jiye Yan

**Affiliations:** 1 Beijing Key Laboratory of Environment Friendly Management on Fruit Diseases and Pests in North China, Institute of Plant Protection, Beijing Academy of Agriculture and Forestry Sciences 107624, Beijing, China; Michigan State University, East Lansing, Michigan, USA

**Keywords:** Botryosphaeriaceae, core effector genes, evolution, *Lasiodiplodia*, super pan-effectome

## Abstract

Effectors are the relatively rapidly evolving genes in fungal phytopathogens. Elucidating the conservation and diversity of effectors is essential to understand the infection mechanisms of phytopathogens. Botryosphaeriaceae encompasses woody host pathogens causing significant economic loss worldwide. However, the pathogenicity mechanisms of these species remain poorly understood. In this study, we comparatively analyzed the effectomes of 25 Botryosphaeriaceae species to characterize the evolutionary dynamics of effector genes at the family level. We identified 56–183 candidate secreted effector proteins (CSEPs) across each of these species. Gene gain events occurred both before and after Botryosphaeriaceae diverged into different genera, while gene loss at the species level has played a prominent role in shaping the effector repertoire. Through pan-effectome analysis, conserved and diversified CSEP families were identified in Botryosphaeriaceae, with the number of conserved CSEP families significantly lower than that of diversified CSEP families. Horizontal gene transfer (HGT) analysis revealed that conserved CSEPs are likely inherited through vertical transmission, whereas many genes from the diversified CSEP families appear to have been acquired through HGT. Conserved CSEPs exhibit earlier expression onset and maintain longer expression duration compared to diversified CSEPs during host infection, and they can suppress plant immunity.

**IMPORTANCE:**

Our results provide compelling evidence for the existence of conserved candidate secreted effector proteins (CSEPs) within the Botryosphaeriaceae family, which may play pivotal roles in woody plant infection. These findings not only deepen our understanding of effector evolution in fungal pathogens but also lay a foundational framework for developing targeted strategies to mitigate the impact of Botryosphaeriaceae-related diseases in woody crops.

## INTRODUCTION

Through prolonged coevolutionary processes, pathogens have developed a variety of sophisticated strategies to suppress plant resistance and invade hosts, with the secretion of effector proteins being one of the most well-known mechanisms (1–3). Effector proteins are typically small secreted proteins containing fewer than 300 amino acids and characterized by their cysteine-rich composition, signal peptide presence, and the absence of transmembrane domains (4). They effectively modulate host physiological processes, such as signal transduction pathways, metabolic activities, and defense mechanisms to create conditions favorable for pathogen infection (5–9).

Effector genes in pathogens undergo intense evolutionary pressure to overcome plant immune defenses. As plants continuously evolve new R genes to recognize and defend against specific effector proteins, pathogens respond to rapid change by inducing high-frequency mutations in their effector genes. These mutations generate new alleles to evade recognition by plant resistance proteins (10–12). Alternatively, they may escape plant recognition through gene loss. For instance, the absence of the effector gene Nip1 in *Rhynchosporium secalis* is the sole mechanism for infecting barley cultivar Rrs1 (13). Moreover, horizontal gene transfer (HGT) significantly contributes to the ability of pathogens to evade plant immune responses. Different pathogens exchange genes through horizontal gene transfer, which enriches the gene pool and offers more opportunities for the evolution of effector proteins (14–16). For example, *Pyrenophora tritici-repentis* acquired the *toxA* effector gene from *Stagonospora nodorum*, significantly enhancing its virulence and posing a severe threat to global wheat yields (17). These diverse evolutionary mechanisms may result in low sequence similarity among effector proteins, with most being diversified and prone to variation (18).

Botryoshaeriaceae family (Botryosphaeriales, Dothideomycetes, and Ascomycota) is a significant group of opportunistic fungal pathogens in agriculture and forestry (19, 20). They have a wide host range, causing various diseases including apple ring rot (21), peach gummosis (22), *Diplodia* shoot blight (23), pitaya canker (24), citrus stem-end rot (25), cassava leaf blight (26), and Botryosphaeria dieback (27), resulting in severe economic losses. Despite their global importance as pathogens, research on the pathogenic mechanisms of these species remains limited, particularly regarding variations across higher taxonomic levels. Currently, research on effector proteins of Botryosphaeriaceae species primarily focuses on a single species. For example, Zhang et al. identified multiple candidate effector proteins in *Botryosphaeria dothidea* and experimentally validated that they can suppress programmed cell death (PCD) triggered by elicitors INF1, MKK1, and NPK1 (28). Furthermore, Wang et al. identified additional candidate effectors in *B. dothidea* that not only interfere with plant PCD but also regulate hydrogen peroxide accumulation (29). In *Lasiodiplodia theobromae*, the effector LtCSEP1 was found to suppress Bax-triggered PCD and inhibit immune responses in *Nicotiana benthamiana* (30). Moreover, it has been shown that the effector LtCre1 targets the glucose signaling protein VvRHIP1 in grapevines, leading to the suppression of host immune responses (31).

With the growing demand for sustainable fungicides, targeting conserved effector proteins is emerging as a promising eco-friendly strategy. Recently, the compound FY21001, designed by Liu et al., has shown the competitive binding to the conserved effector protein MoErs1 of *Magnaporthe oryzae* and exhibits significant control efficacy against rice blast disease (32). While notable progress has been made in effector-mediated control in rice blast, research on other fungal diseases remains limited. While Botryosphaeriaceae species are widely acknowledged as woody plant pathogens, it remains unclear whether conserved effector proteins exist across the family that contribute to their infection capabilities.

This current study aims to understand and elucidate the evolutionary patterns of the effector proteins and identify conserved effector proteins in Botryosphaeriaceae family using super pan-effectome. Herein, the pan-effectome of 25 species within the Botryosphaeriaceae family was analyzed. Functions of the conserved effector proteins were further clarified through transcriptomic analysis and biochemical as well as molecular biological experiments. This study pioneers the application of super pan-effectome techniques to identify and analyze family-level conserved effector proteins, providing a reference for similar studies in other pathogens.

## MATERIALS AND METHODS

### Genome sequence collection

The whole-genome sequences of 25 fungal species from Botryosphaeriaceae were downloaded from GenBank (https://www.ncbi.nlm.nih.gov/), belonging to seven genera: *Botryosphaeria*, *Diplodia*, *Dothiorella*, *Macrophomina*, *Neoscytalidium*, *Neofusicoccum*, and *Lasiodiplodia* (Table S1). The NCBI BioProject accession numbers can be found in Table S1. We included only representative genomes from GenBank with a BUSCO score greater than 95% in our analysis.

### Genome annotation and gene prediction

For each genome, *de novo* prediction of genomic repeat sequences was performed using RepeatModeler v1.0.11 (33) to establish a repeat sequence library. Based on the repeat sequences library, RepeatMasker v4.0.9 (34) was utilized to annotate the repeat sequences in the genome. Subsequently, gene prediction was conducted using Braker2 in conjunction with software such as GeneMark-EP+ and AUGUSTUS based on the repeat sequences annotation results (35). During the execution of Braker2, the OrthoDB fungal protein database v10 was employed for homology-based prediction. The completeness of each genome was evaluated using BUSCO v3.0.2 (36) software in conjunction with the fungi_odb10 database.

### Effector protein prediction and domain analysis

To identify candidate effector proteins, N-terminal signal peptides were predicted using SignalP (37) to select sequences containing N-terminal signal peptides. Simultaneously, transmembrane domains were predicted using TMHMM to predict transmembrane regions, and proteins containing transmembrane domains were removed. Protein subcellular localization analysis was performed using TargetP (38), and proteins localized to organelles were removed. For the remaining proteins, candidate effector proteins were identified using EffectorP 2.0 (39). The HMMsearch command of the hmmer3.4 software was used to analyze the conserved domains of candidate effector protein sequences (40). Effector protein-related conserved motifs CFEM (PF05730), LysM (PF01476), LysM (PF07897), DPBB_1 (PF03330), cutinase (PF01083), and chitin-binding (PF00187) were downloaded from the Pfam website (http://pfam.xfam.org/) and used as query sequences. When running HMMsearch, the *E* value was set to 10^–3^.

### Phylogenomic analyses

Protein sequences containing premature termination codons were extracted and removed from 25 genomes. Gene family analysis of the proteins was performed using OrthoFinder to extract single-copy genes (41). A rooted species tree was constructed using *Aplosporella prunicola* and *Saccharata proteae* as outgroups. First, homologous genes were aligned using MAFFT v7.313 (42), and poorly aligned regions were removed using trimAl (43). The aligned sequences were then concatenated. Finally, a phylogenetic tree was constructed using the maximum likelihood method IQ-Tree v1.6.12 with the parameter set to -m MFP to automatically test and select the optimal substitution model (44). Standard non-parametric bootstrap (-bb 1000) was used to evaluate branch support. The phylogenetic tree was visualized using iTOL (45). Based on the Time Tree website (https://timetree.org/), the fossil divergence time of the species *A. prunicola* and *S. proteae* was 57 million years (Mya). The MCMCTREE program in Paml was used to estimate species divergence times (46), with the root crown time of Botryosphaeriaceae set to around 61 Mya (47). This method was run twice to determine the convergence of the results. The results were visualized using FigTree (https://tree.bio.ed.ac.uk/software/figtree/). As described above, OrthoFinder was used to cluster effector proteins from 25 species, and protein sequences from each gene family were extracted. These sequences were aligned using MAFFT, and poorly aligned regions were removed using trimAl. Finally, IQ-Tree v1.6.12 was used to construct a gene tree of effector proteins.

### Gene family expansion and contraction analysis

CAFÉ v5 (Computational Analysis of Gene Family Evolution) was used to analyze the expansion and contraction of gene families (48). This program uses birth and death processes to simulate gene gain and loss in a specified phylogenetic tree, illustrating the phylogenetic history. In the genomes of 25 species, 17,115 protein gene families and 342 effector protein gene families were used for the expansion and contraction analysis of whole-genome protein gene families and effector protein gene families, respectively. Additionally, the obtained phylogenetic time tree was used to analyze the evolution of gene families. Gene functional annotation was performed using Interproscan v5.47-82.0 (49). Based on the GO (Gene Ontology) annotation results, functional enrichment analysis was conducted using the hypergeometric test method. The *P* value was corrected using the Benjamini-Hochberg method to obtain FDR values, and an FDR value < 0.05 was used as the threshold for functional enrichment analysis.

### Selection pressure analysis

Pairwise comparisons of protein sequences from 25 Botryosphaeriaceae fungi were performed using BlastP, with an *E* value set to 10^−5^. For each gene, the best-matching sequence from other species was selected, and sequence alignment was performed using MAFFT. Then, Pal2Nal v14 was used to convert the protein multiple sequence alignment into a CDS alignment (50). The APE package in R was used to generate a tree file corresponding to the species in the CDS sequence (51). The codeml command in Paml was used to calculate the Ka/Ks ratio of gene families (52).

### Horizontal gene transfer analysis

The NCBI nr database (Non-Redundant Protein Database, 2023.11.10) was downloaded, and the protein sequences of 25 strains were compared with the nr database using Diamond BLASTp method, with an *E* value set to e^−10^ (53). For each protein gene, the Alien Index (AI) score of the gene was calculated based on the diamond results, using species within Dothideomycetes as closely related organisms and species outside Dothideomycetes as distantly related species (54). This method is used to identify potential horizontal gene transfer (HGT) events by marking genes whose alignment results with distant organisms are significantly better than those with closely related organisms. The higher the AI value, the greater the likelihood of horizontal gene transfer. Genes with an AI value greater than 10 were selected (55), and the top 500 optimal alignment sequences (only one record per species) were chosen based on the diamond blastp alignment results for MAFFT sequence alignment. After removing poorly aligned regions, sequences shorter than 50 amino acids were discarded. The potential HGT genes were further identified through the following steps: initially, FastTree was employed to construct phylogenetic trees with parameters -lg -spr 4 -mlacc 2 -slownni (56), followed by preliminary HGT screening with NestedIn software, which searches for the nested position of query sequences among sequences from donor groups containing at least two confident nodes (57). We only considered species from distantly related donors outside Dothideomycetes, including intra-kingdom donors of non-Dothideomycetes fungi and inter-kingdom donors of viruses, archaea, bacteria, animals, and plants. Secondly, based on the initial screening results, IQ-Tree was run with an automatically selected best-fit model and standard non-parametric bootstrap (-bb 1000) (44). The results were then filtered again using NestedIn. The horizontal gene transfer gene trees were visualized with iTOL (45). Finally, manual verification was performed.

### Transcriptome analysis

The *L. theobromae* transcriptome sequencing data were obtained from a study published by Peng et al. (55). These data include transcriptome sequencing data from lesions on annual grape (*Vitis vinifera* cv. “Summer Black”) branches inoculated with *L. theobromae in vitro* after 12, 24, and 48 h at 25°C, as well as transcriptome sequencing data from mycelium cultured on PDA medium after 12, 24, and 48 h.

After filtering low-quality sequences using fastp (58), the transcriptome sequencing data were mapped to the *L. theobromae* genome using Hisat2 software (59). Gene differential expression analysis was performed using edgeR (60) software, and *P* values were corrected using the Benjamini-Hochberg method. Genes were considered differentially expressed when the *P* value was < 0.05 and |log_2_ FC| > 0.585.

### Cell-death suppression assay in *Nicotiana benthamiana*

The *N. benthamiana* plants were grown in a controlled growth chamber at 25°C with 60% relative humidity under a 16 h light/8 h dark photoperiod. The CDS coding sequence (CDS) of core Botryosphaeriaceae effectors without the signal peptide was cloned and introduced into the binary vector pGD-GFP using NEBuilder HiFi DNA Assembly Cloning Kit (NEB, Ipswich, MA, USA) with primers (Table S2) (61). All vectors constructed were verified by sequencing and then transformed into *A. tumefaciens* strains GV3101. The empty pGD-GFP vector was used as a control. Agrobacterium strains harboring the corresponding vectors were used to infiltrate into the leaves of 4-week-old *N. benthamiana* plants using a needle-free syringe. Subsequently, a second infiltration with agrobacteria carrying the BAX gene was performed 12 h after the initial infiltration. Plants were maintained under the same environmental conditions throughout the experiment. Five days after the final injection, photographs were taken to record leaf cell death symptoms. The extent of BAX-induced cell death was determined based on ion leakage in tobacco leaf disks after Agrobacterium injection. Ion leakage was measured 3 days after BAX infiltration. This experiment was repeated three times.

### Gene expression analysis

Total RNA was isolated from freshly harvested tobacco leaf tissues. Briefly, leaf samples were immediately frozen in liquid nitrogen and thoroughly ground into a fine powder using a chilled mortar and pestle. The powdered tissue was then directly mixed with the lysis buffer provided in the EasyPure Universal Plant Total RNA Kit (TransGen Biotech, Beijing) and processed according to the manufacturer’s instructions. The RNA concentration was determined using a NanoDrop 2000c spectrophotometer (Thermo Scientific, Waltham, MA, USA). A total of 3 μg of the isolated RNA was reverse-transcribed into cDNA using the Superscript III First-Strand Synthesis SuperMix Kit (Invitrogen). Reverse transcription-PCR (RT-PCR) was conducted using Takara Premix LA Taq (TaKaRa, Dalian, China) on the C1000 Touch thermal cycler PCR System (Applied Biosystems, Foster City, CA, USA). The *Actin* gene from *N. benthamiana* was used as the internal reference. All primers are listed in Table S2.

### Generation of the *Lasiodiplodia theobromae* overexpression transformants

To construct overexpression vectors, the full-length coding sequences (CDS) of the effector genes Lt-g1149 and Lt-g3745 were amplified using gene-specific primers (Table S3) and subsequently sub-cloned into a modified pBluescript II KS vector using In-Fusion HD Cloning Kits (TaKaRa). For protoplast transformation of *L. theobromae*, 150 μL of protoplasts was mixed with 2 μg of linearized recombinant plasmid in a 50 mL centrifuge tube. The volume was adjusted to 300 μL with STC buffer, and the mixture was incubated on ice for 20 min. Subsequently, 2 mL of PTC solution was added dropwise, followed by an additional 20-min incubation on ice. After adding 25 mL of pre-chilled STC solution and inverting the samples, the samples were centrifuged at 2,000 × *g* at 4°C for 15 min. The supernatant was discarded, and the pellet was resuspended in 3 mL of LR medium. The transformed protoplasts were then incubated at 28°C for 12–18 h. Finally, 10 mL of SR regeneration medium was added, and the mixture was thoroughly blended and poured onto plates. After solidification, the agar surface was overlaid with 15 mL of 1.5% water agar supplemented with the appropriate selection antibiotic. Positive transformants were screened by PCR and validated by qRT-PCR using gene-specific primers (Table S3).

### Pathogen inoculation assays

The vectors were transformed into the Agrobacterium GV3101 strain and transiently expressed in tobacco leaves grown for 4 weeks. Detached tobacco leaves were inoculated on their abaxial surface with a spore suspension of *L. theobromae* at a concentration of 1 × 10^6^ conidia∙mL^−1^. The samples were placed in an inoculation chamber at 26°C and 90% relative humidity under a 16 h light/8 h dark photoperiod. Photographs were taken, and lesion lengths were measured 5 days later. The green fluorescence was observed using a confocal scanning laser microscope (LSM900, Carl Zeiss, Oberkochen, Germany) at 48 h post-infiltration, with pGD-GFP as the control.

Healthy green grapevine shoots from 1-year-old *Vitis vinifera* var. “Summer Black” were used for the inoculation assay. A sterile 4 mm cork borer was used to remove the phloem tissue and the wounded site was inoculated with a conidial suspension (1 × 10^6^ spores mL^−1^) prepared in a 0.02% Silwet L-77 solution. The inoculation point was wrapped with sealing film to maintain humidity. The shoots were planted in nutrient pots filled with sterilized vermiculite and transferred to a greenhouse maintained under controlled conditions with a 16 h light/8 h dark photoperiod, temperatures of 25°C/18°C (day/night), and 50% relative humidity. The vermiculite was kept moist throughout the experiment. Disease development on the shoots was monitored and recorded 5–7 days post-inoculation.

## RESULTS

### Identification and characterization analysis of genome-wide candidate effectors

**Fig 1:**
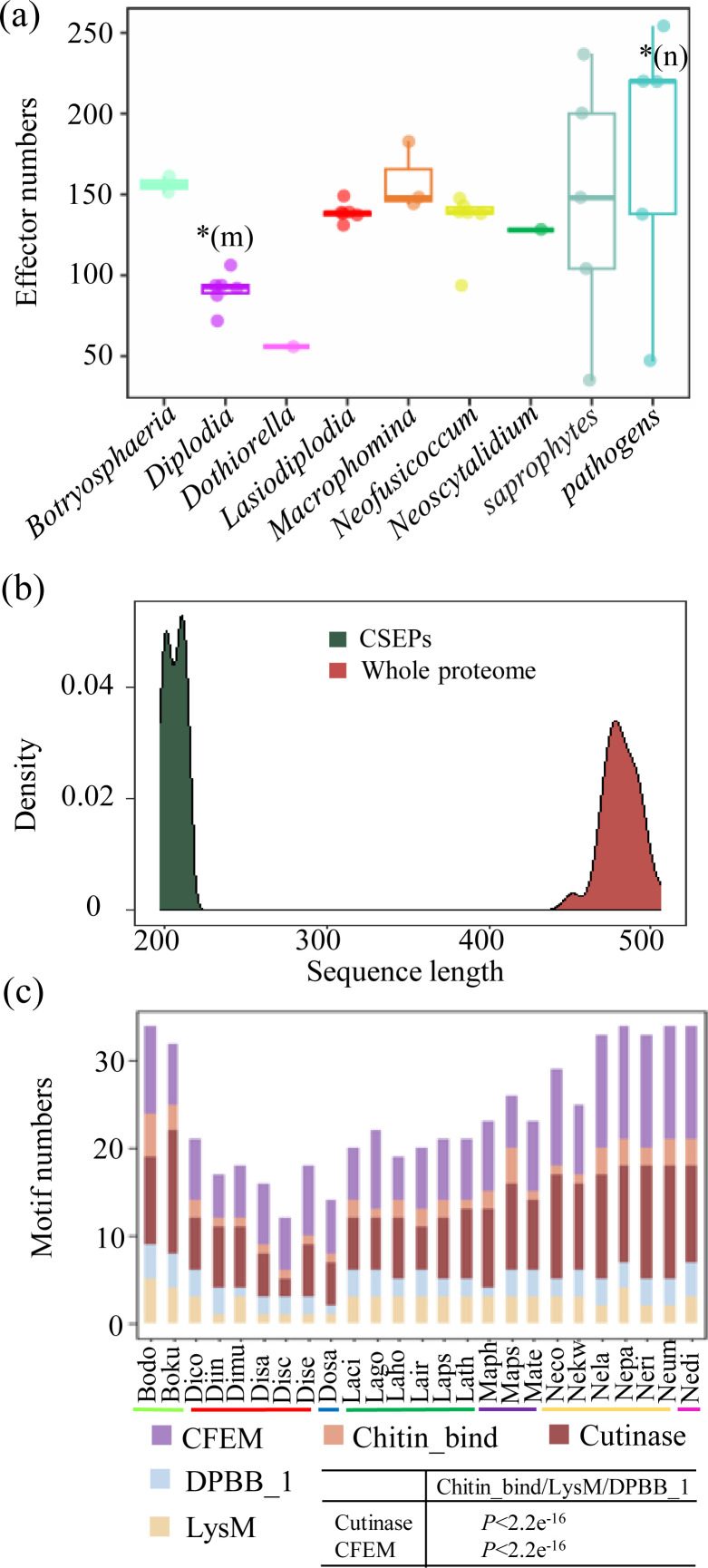
Sequence features of Botryosphaeriaceae candidate secreted effector protein (CSEP) genes. (**a**) Number of CSEPs in Botryosphaeriaceae, pathogenic fungi, and saprophytic fungi. Two types of statistical analyses were performed: (i) comparisons among genera within Botryosphaeriaceae, and (ii) comparisons between Botryosphaeriaceae fungi and saprophytic fungi, as well as between Botryosphaeriaceae fungi and pathogenic fungi. In the intra-Botryosphaeriaceae comparisons, *Dothiorella* and *Neoscytalidium* were excluded from statistical analyses due to a sample size of one but are shown in the figure for comparison. Asterisks indicate significant differences based on Dunn’s post hoc tests with Benjamini-Hochberg correction. *(m) indicates a significant difference between *Diplodia* and the genera *Botryosphaeria*, *Lasiodiplodia*, *Macrophomina*, and *Neofusicoccum* based on Dunn’s post hoc tests with Benjamini-Hochberg correction (*P* < 0.05). *(n) indicates a significant difference between Botryosphaeriaceae fungi and pathogenic fungi (*P* < 0.05). (**b**) Length distribution of sequences in CSEPs and whole-genome proteins. (**c**) Association of CSEP genes with conserved motifs commonly found in plant pathogenic fungi. Pairwise comparisons among groups were performed using chi-square tests. Only significant comparisons are shown. *Y*-axis: number of motifs per species.

Genome sequences of 25 species in Botryosphaeriaceae, representing seven distinct genera (*Botryosphaeria*, *Diplodia*, *Dothiorella*, *Macrophomina*, *Neoscytalidium*, *Neofusicoccum*, and *Lasiodiplodia*), were downloaded from GenBank. The high quality of these assemblies was supported by BUSCO completeness scores exceeding 95.40% across all genomes, ensuring a robust foundation for subsequent comparative analyses (Table S1). Gene models were generated using BRAKER, and candidate secreted effector proteins (CSEPs) were predicted using a combination of SignalP, TMHMM, TargetP, and EffectorP. In total, 3,151 CSEPs were identified across the 25 species. The number of CSEPs differed significantly among genera (Kruskal-Wallis test, *P* = 0.0024). Post hoc Dunn’s tests with Benjamini-Hochberg correction revealed that *Diplodia* species contained significantly fewer CSEPs than species of *Botryosphaeria*, *Lasiodiplodia*, *Macrophomina*, and *Neofusicoccum* (BH-adjusted *P* < 0.05), whereas no significant differences were detected among the latter four genera (Fig. 1a; Table S1). In addition, the overall number of CSEPs in Botryosphaeriaceae was comparable to that observed in saprophytic fungi but slightly lower than that in pathogenic fungi (Fig. 1a). We further explored the relationship between CSEP number and host range across species. No significant correlation was detected (Fig. S1), indicating that variation in effector gene number alone does not necessarily reflect host range differences. The length of CSEPs was significantly shorter, with lengths ≤300 amino acids, compared to whole-genome proteins, which typically ranged from 400 to 500 amino acids (Fig. 1b). Effector proteins of plant pathogenic fungi possess an array of commonly conserved motifs (62), such as Chitin_bind, Cutinase, LysM, DPBB_1, and CFEM motifs, to mediate their pathogenic processes (63–65). CSEPs with these common motifs were characterized using hmmsearch. In Fig. 1c, all investigated species harbored all five conserved motifs. Pairwise comparisons revealed that CFEM and Cutinase motifs were significantly more abundant than Chitin_bind, DPBB_1, and LysM motifs (chi-square test, *P* < 2.2 × 10^−16^), whereas no significant differences were detected among Chitin_bind, DPBB_1, and LysM motifs. CFEM proteins are a class of fungal-specific proteins located on the extracellular membrane (66, 67), and effector proteins that contain the CFEM motif play a crucial role in fungal virulence (68, 69). Cutinases are enzymes that degrade cutin components in the plant cuticle layer (70, 71). Also, we could observe differences in the number of motifs among species from different genera. Species within the genera *Botryosphaeria* and *Neofusicoccum* exhibited a significantly higher number of motifs compared to those in other genera, whereas species in *Diplodia* showed a notably lower number of motifs than the rest. This high divergence of motifs among different genera may be associated with the host infection range as well as the co-evolution between hosts and pathogens (72, 73).

### Effector gene family expansion and contraction in Botryosphaeriaceae

**Fig 2:**
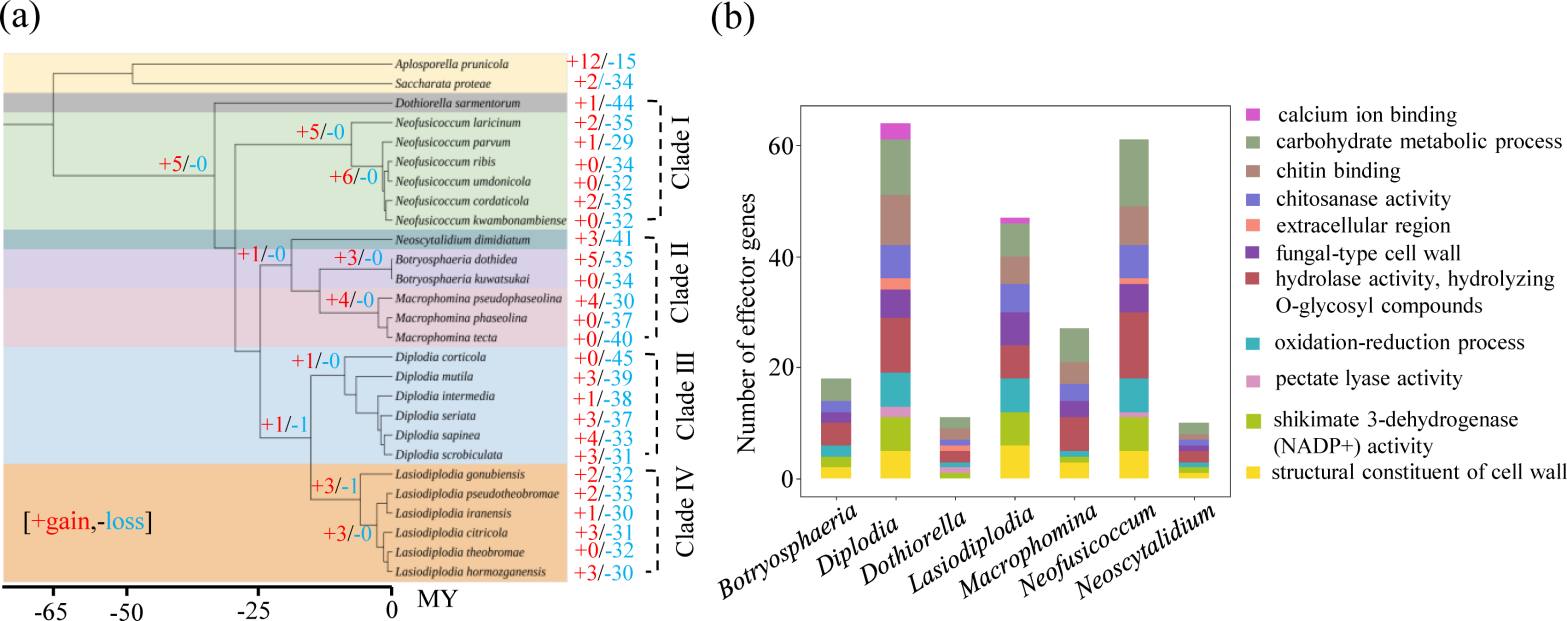
Gene family expansion and contraction in CSEP genes of Botryosphaeriaceae. (**a**) Gene family gain and loss analysis of CSEPs in Botryosphaeriaceae. (**b**) Functional analysis of contracted effector gene families at the species level, categorized by different genera.

Phylogenetic analysis of 25 Botryosphaeriaceae fungi revealed their division into four distinct evolutionary lineages, as shown in Fig. 2a. Clade I encompassed the genera *Dothiorella* and *Neofusicoccum*, which diverged approximately 33.75 million years ago (Mya). Clade II comprised *Neoscytalidium*, *Botryosphaeria*, and *Macrophomina*, sharing a common ancestor that dates back to around 19.12 Mya. In contrast, Clades III and IV each represented single genera: *Diplodia* with a divergence time of ~9.01 Mya (Clade III) and *Lasiodiplodia* with a divergence time of ~6.06 Mya (Clade IV). The broad temporal range of these divergences, spanning from ~6.06 to 33.75 Mya, highlights the considerable evolutionary diversity and complexity within the Botryosphaeriaceae family.

Through the application of OrthoFinder software, a comprehensive analysis was conducted on 324,339 protein sequences and 3,151 CSEPs sequences. This process successfully identified 17,115 protein gene families and 342 effector gene families. Subsequent gene family expansion and contraction analyses were conducted based on a time-calibrated species tree, revealing distinct patterns between genome-wide protein gene families and effector gene families. For genome-wide protein gene families, the most recent common ancestor (MRCA) of the four clades (I–IV) experienced a prominent pattern of gene family expansion, while both expansions and contractions were observed within each branch during subsequent divergence into various genus-level ones (Fig. S2). The effector gene family also predominantly underwent expansions in the MRCA of the four clades. However, the difference was that the process of divergence at the genus-level branches was mainly characterized by the continuous gene family expansions within each branch (Fig. 2a). Additionally, the five ancient gene families experienced gene gain in the MRCA of the seven genera, including a pectin lyase gene family OG0000000. The parent branches of *Neofusicoccum*, *Botryosphaeria*, *Macrophomina,* and *Diplodia* experienced gene gain (five, three, four, and one family, respectively). On the other hand, one family had gene loss while three families evolved through gene gain of the parent branch of *Lasiodiplodia*. Functional annotation was conducted on the genes gained after family divergence but prior to speciation, and it was found that these genes were mainly enriched in functions related to pectate lyase activity, extracellular region, and calcium ion binding.

Effector gene family contraction events surpassed expansion events after speciation. Species such as *N. ribis*, *N. umdonicola*, *B. kuwatsukai*, *M. phaseolina*, *M. tecta*, *D. corticola*, and *L. theobromae* showed only gene loss (+0/−34, +0/−32, +0/−34, +0/−37, +0/−40, +0/−45, and +0/−32, respectively). The number of families with gene loss in species such as *D. sarmentorum*, *N. laricinum*, *N. dimidiatum*, *B. dothidea*, *M. pseudophaseolina*, *D. mutila*, and *L. citricola* was significantly higher than the number of families with gene gain (+1/−44, +2/−35, +3/−41, +5/−35, +4/−30, +3/−39, and +3/−31, respectively) (Fig. 2a). However, the gene gain and loss situations within the whole-genome protein gene families of the aforementioned species were as follows: +819/−1,216, +303/−1,107, +484/−1,048, +977/−894, +305/−940, +496/−921, and +211/−815, respectively (Fig. S2). Compared with the whole-genome protein gene families, the degree of gene loss of effector gene families was more pronounced at the species level. This suggested that, although gene family expansions in effector genes were identified, the loss of effector proteins was a common phenomenon at the species level, particularly in recent evolutionary history. Gene ontology (GO) analysis showed that contracted families within different genera were enriched in carbohydrate metabolism, chitin binding, and hydrolase activity, particularly in the hydrolysis of O-glycosyl compounds (Fig. 2b).

### Super pan-effectome analysis of effectors in Botryosphaeriaceae

**Fig 3:**
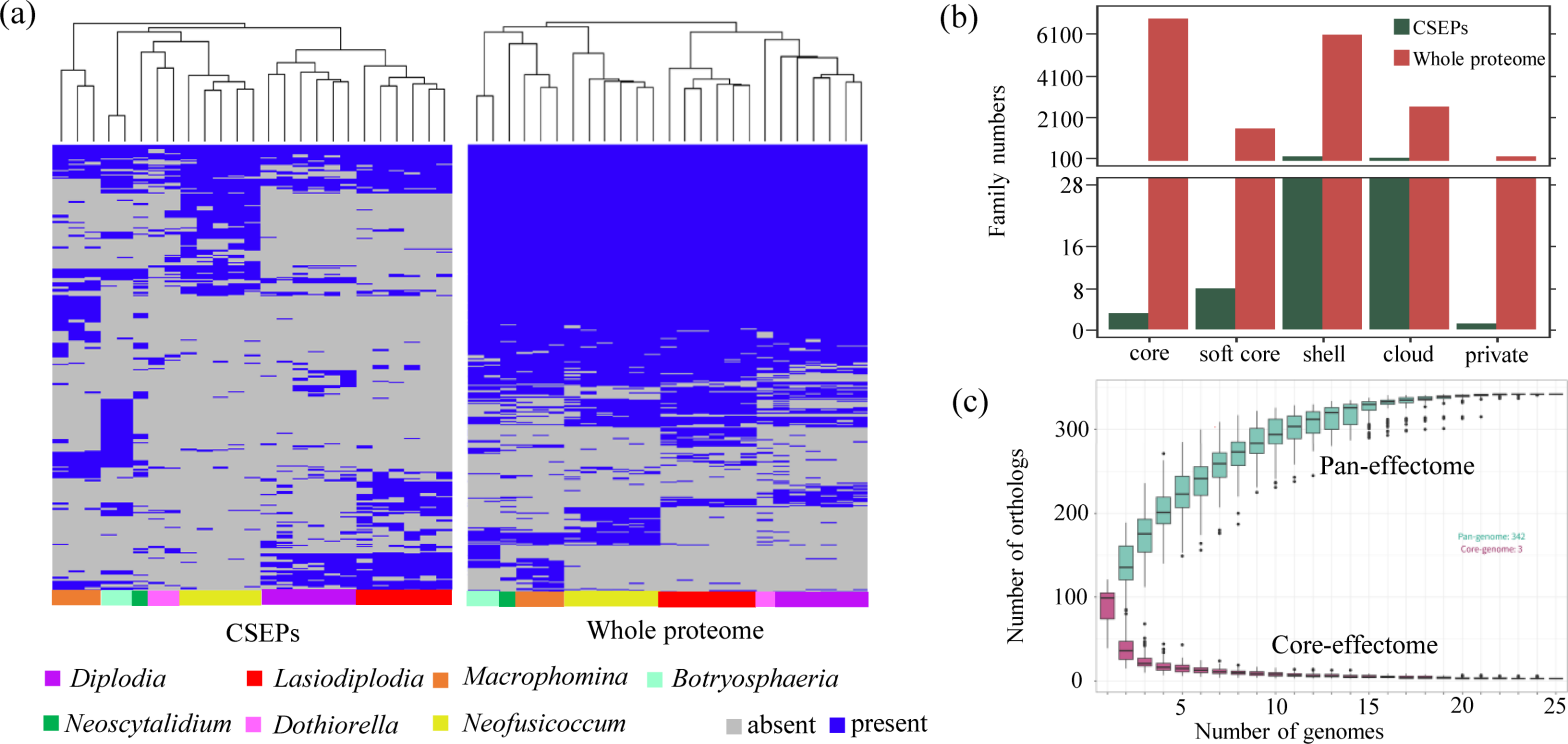
Pan-effectome analyses of Botryosphaeriaceae. (**a**) Presence or absence of members from effector gene families or protein-coding gene families across 25 Botryosphaeriaceae species. (**b**) Comparative analysis of gene family numbers across different categories. (**c**) Changes in the number of pan-effectome and core-effectome as the genome numbers increase.

The pan-genome represents the complete collection of genomic information from all individuals within a species, while the pan-effectome comprises the collective effector protein information, which could provide us with more comprehensive understanding (74, 75). Through super pan-effectome analysis of 25 species in Botryosphaeriaceae, it was observed that, in comparison to the pan-proteome, the conservation among gene family members within the pan-effectome is extremely low. There are very few CSEPs conserved at the genus level across the Botryosphaeriaceae, and even fewer conserved at the family level (Fig. 3a).

The pan-effectome consisted of core and dispensable categories. Specifically, it comprised 3 core categories shared by all species, 8 soft-core categories present in more than 95% of the species, 191 shell categories occurring in 15–95% of the species, 139 cloud categories present in fewer than 15% of the species, and 1 private category unique to a single species. In contrast, the pan-proteome contained 6,845 core categories, representing approximately 40% of all protein gene families (Fig. 3b). Notably, core categories accounted for only 0.88% of the pan-effectome, highlighting a dramatically lower level of conservation relative to the pan-proteome. The pronounced dominance of dispensable categories within the pan-effectome indicates extensive diversification and rapid turnover of effector gene families during the evolution of Botryosphaeriaceae. Analysis of pan-genome accumulation curves further supported these observations. Saturation of the pan-proteome was reached after the inclusion of approximately 16 genomes, whereas the core-proteome continued to expand until around 23 genomes were incorporated (Fig. S3). By comparison, the pan-effectome reached saturation after the integration of roughly 18 genomes, while the core-effectome stabilized much earlier, following the addition of only about 5 genomes (Fig. 3c). Together, these results indicate that, although effector repertoires are highly dynamic and species-specific, their overall diversity is constrained. Beyond a certain number of genomes, the inclusion of additional species did not result in further expansion of the pan-effectome, suggesting that effector gene families have finite evolutionary limits rather than expanding indefinitely.

Functional annotations were carried out on three core categories. Two of these were respectively annotated as Pectate lyase and Peptidase inhibitor family, whereas the function of the remaining family is still unknown. Next, the Ka/Ks ratio was employed to investigate the selective pressures acting on various categories of effector gene families throughout their evolutionary trajectory. Compared to shell and cloud categories, core and soft-core categories have lower Ka/Ks values, suggesting that the latter are under more purifying selection pressure. This pattern aligns with genome-wide analyses, where similar trends were observed (Fig. S4).

### Pathogens acquire effector genes through horizontal gene transfer

**Fig 4:**
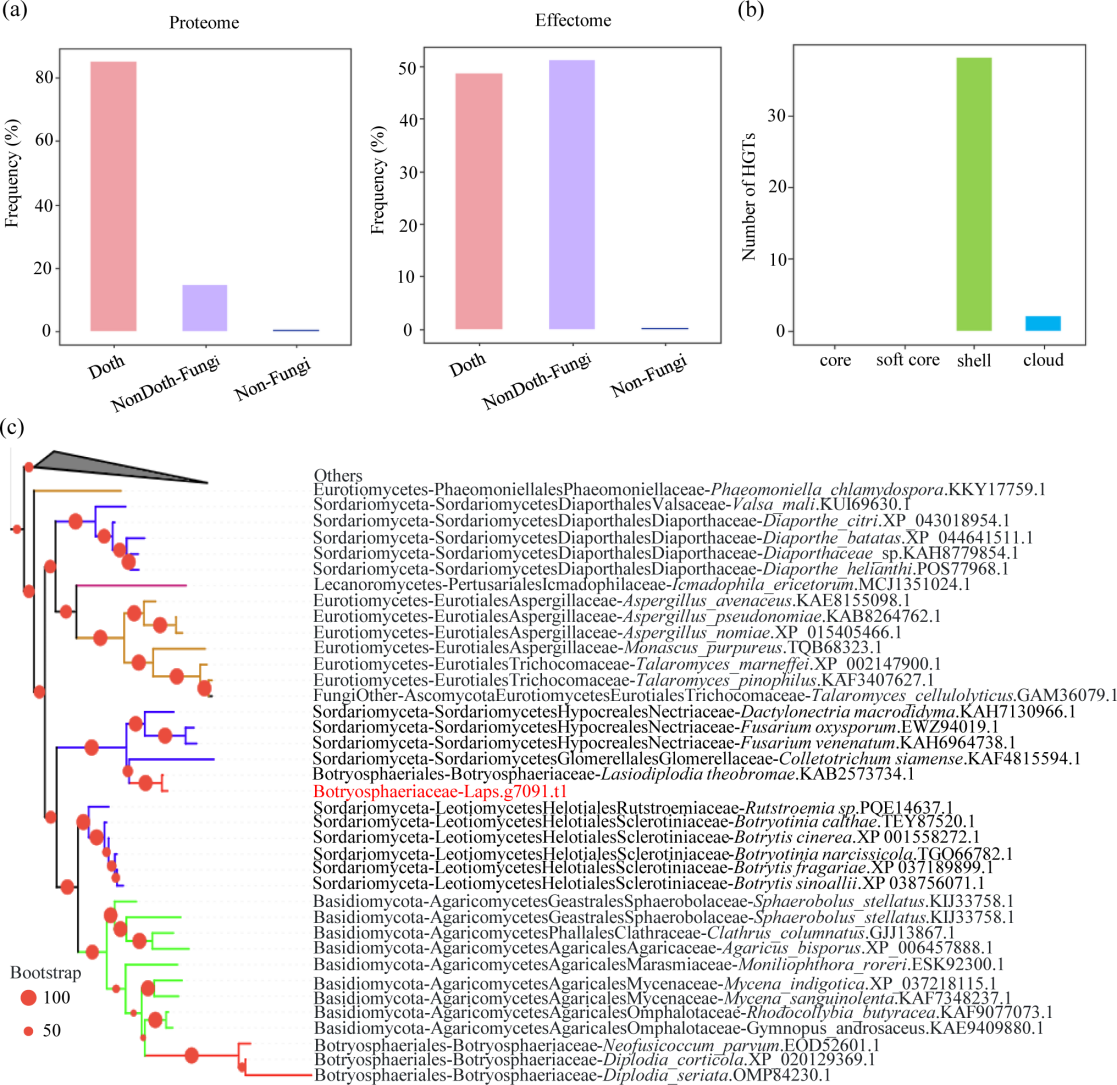
Horizontal gene transfer (HGT) analysis of Botryosphaeriaceae CSEPs. (**a**) Taxonomic distribution of the closest species in the top BLAST hits of Botryosphaeriaceae proteins against the NR database. (**b**) Comparative analysis of horizontal gene transfer (HGT) events among different effector gene categories. (**c**) An example of horizontal gene transfer of cutinase CSEP gene from *Sordariomycetes* to *L. pseudotheobromae*. Laps.g7091.t1 in *L. pseudotheobromae* was used as the query to build the gene tree. The full tree information can be found in Fig. S10.

To assess whether horizontal gene transfer occurs within the Botryosphaeriaceae, the genome-wide protein sequences and effector sequences of 25 species were blasted against the nr database using Diamond software (53). We then classified each gene into different evolution groups based on the phylogenetic distance of the species to Dothideomycetes from its best-matched non-Botryosphaeriaceae blast hit (Fig. S5). If the best hits are matched in phylogenetically distant species, there is a high probability that these genes have been horizontally transferred. At the genome-wide level, 85.14% of protein-coding genes showed the species of top hits within Dothideomycetes, 14.62% in non-Dothideomycetes but within Fungi, and 0.24% in non-Fungi organisms (Fig. 4a). This distribution suggested that most of the protein-coding genes may have originated from vertical gene transfer. However, CSEPs exhibited a markedly different pattern: 51.15% of top hits were found in non-Dothideomycetes but within Fungi, followed by 48.63% in Dothideomycetes, and 0.22% in non-Fungi organisms (Fig. 4b). These results suggested that CSEP genes may have been acquired through HGT, as the most closely matching sequences in these genes exhibit distant phylogenetic relationships.

For each CSEP gene, the AI score was used to identify potential HGTs. Using Dothideomycetes as closely related organisms and species outside Dothideomycetes as distantly related organisms, we identified 1300 CSEP genes with the AI score of more than 10, suggesting high potential of these genes to be HGTs. For each of these 1,300 genes, we then constructed their phylogenetic trees based on their top 500 blastp hits in the nr database, and used non-Dothideomycetes fungi, plants, animals, bacteria, and viruses as donor groups, to characterize potential HGTs further. Subsequently, after filtering with the NestedIn tool and conducting manual validation for each phylogenetic tree, a total of 40 candidate horizontally transferred genes were ultimately identified (Table S4). These potential HGT CSEP genes were predominantly found within the shell (38 potential HGT genes) and cloud (2 potential HGT genes) categories. Notably, no potential HGT genes were detected in the core or soft-core categories (Fig. 4b). These potential HGT genes are most likely to originate from two fungal groups: Sordariomycetes and Basidiomycota. Specifically, 39 genes were found to have been acquired from Sordariomycetes, while only a single gene could be traced back to Basidiomycota (Fig. S6). The class Sordariomycetes comprises a diverse range of highly virulent pathogens, including well-known species such as *Magnaporthe oryzae*, *Fusarium graminearum*, *F. oxysporum*, and various species of the *Colletotrichum* genus (76). An example of CSEP HGT is illustrated in Fig. 4c, which shows the acquisition of the gene Lp-g7091 from *L. pseudotheobromae* (Botryosphaeriaceae) through transfer from Sordariomycetes (Ascomycota).

### Gene expression analysis of core effector protein gene family

To investigate the transcriptional dynamics of candidate secreted effector proteins (CSEPs) during host infection, we analyzed RNA-seq data from *L. theobromae* infecting *Vitis vinifera* cv. “Summer Black” at 12, 24, and 48 h post-inoculation, with mycelium grown on PDA as the control. Differential expression analysis identified genes as differentially expressed (DEGs) based on adjusted *P* < 0.05 and |log₂FC| > 0.585. The raw transcriptomic sequencing data for *L. theobromae* were filtered to obtain clean reads, which were then aligned to the reference genomes (Table S5). In *L. theobromae*, a total of 11,219 genes were detected to be expressed, including 89 expressed CSEP genes.

**Fig 5:**
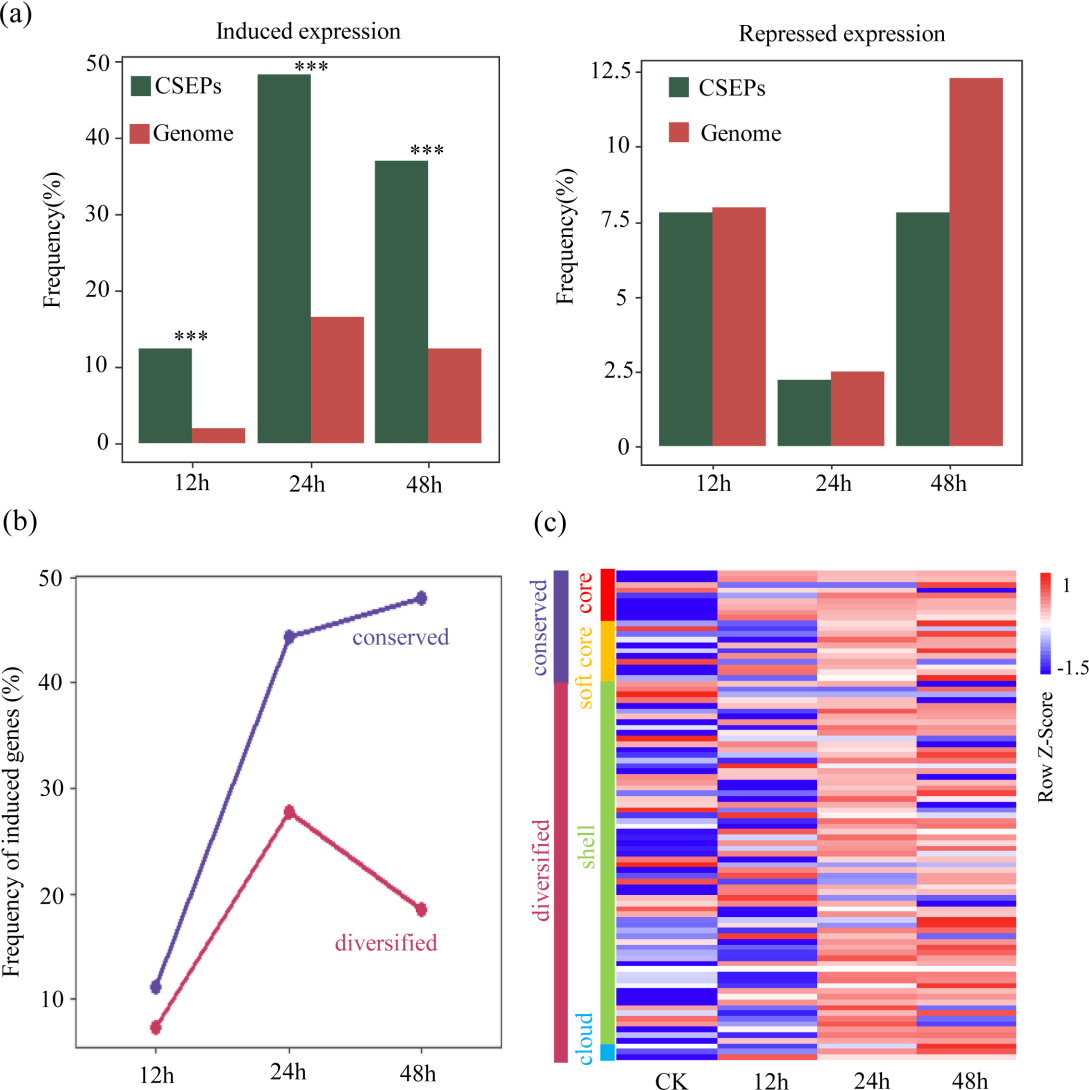
Transcriptome analysis of *Lasiodiplodia theobromae*. (**a**) Proportion of differentially expressed genes (DEGs) among CSEPs compared with non-effector genes at 12, 24, and 48 h post-inoculation. Differentially expressed genes were defined based on adjusted *P* < 0.05 and |log_2_FC| > 0.585. Percentages represent the number of upregulated or downregulated genes relative to the total number of expressed genes in each group. ***: Chi-square test *P* < 0.001. (**b**) Proportion of upregulated CSEPs within different pan-effectome categories (conserved and diversified categories) at each time point. Percentages indicate the number of upregulated CSEPs divided by the total number of CSEPs within each category. “Conserved”: core and soft-core categories are grouped as conserved categories. “Diversified”: shell and cloud categories are grouped as non-conserved categories. (**c**) Heatmap showing expression profiles of individual CSEPs across different pan-effectome categories at 12, 24, and 48 h post-inoculation. Expression levels are represented as FPKM values, scaled by row, with color gradients indicating relative expression intensity. CK: the control group. Panels b and c employ different grouping of effector categories to optimally address distinct analytical questions (see Results for details).

At the genome-wide level, the proportion of upregulated DEGs among CSEPs was consistently higher than that observed for non-effector genes at all examined time points, whereas the proportion of downregulated DEGs was lower (Fig. 5a), indicating a preferential induction of effector genes during host infection. To further examine whether transcriptional responses differed among effector conservation categories, CSEPs were grouped into conserved (core and soft-core) and diversified (shell and cloud) categories. For each category, the percentage of DEGs was calculated as the number of upregulated CSEPs divided by the total number of CSEPs within that category. At 12 h post-inoculation, conserved categories showed a higher proportion of upregulated genes (11.1%) compared with diversified categories (7.4%) (Fig. 5b). At 24 h and 48 h, the proportion of upregulated genes in conserved categories further increased to 44.4% and 48.1%, respectively, while diversified categories also exhibited elevated proportions at later stages (27.7% and 18.5%, respectively). Besides, we further visualized expression patterns using heatmaps based on FPKM values of individual CSEPs across different time points and pan-effectome categories (Fig. 5c). These heatmaps revealed that CSEPs from core categories were transcriptionally activated earlier during infection and maintained relatively high expression levels for a longer duration, whereas CSEPs from diversified categories tended to show delayed and more variable expression patterns. Together, these results indicate that conserved CSEPs are not only preferentially induced during host infection but also exhibit earlier and more sustained expression compared with diversified CSEPs.

### Core and horizontal transferred effectors of Botryosphaeriaceae enhance plant susceptibility

We further investigated the functions of Botryosphaeriaceae CSEP genes in plant-pathogen interaction, and five genes were selected from core categories. These included Bd-g9564, Bd-g5927, Lt-g3745, and Lt-g1149 from the OG0000000 gene family annotated as pectin lyase, and Lt-g11619 from the OG0000001 gene family annotated as a peptidase inhibitor. Additionally, one CSEP gene annotated as a cutinase, Lp-g7091, was selected as a horizontal transfer gene from dispensable gene families.

**Fig 6:**
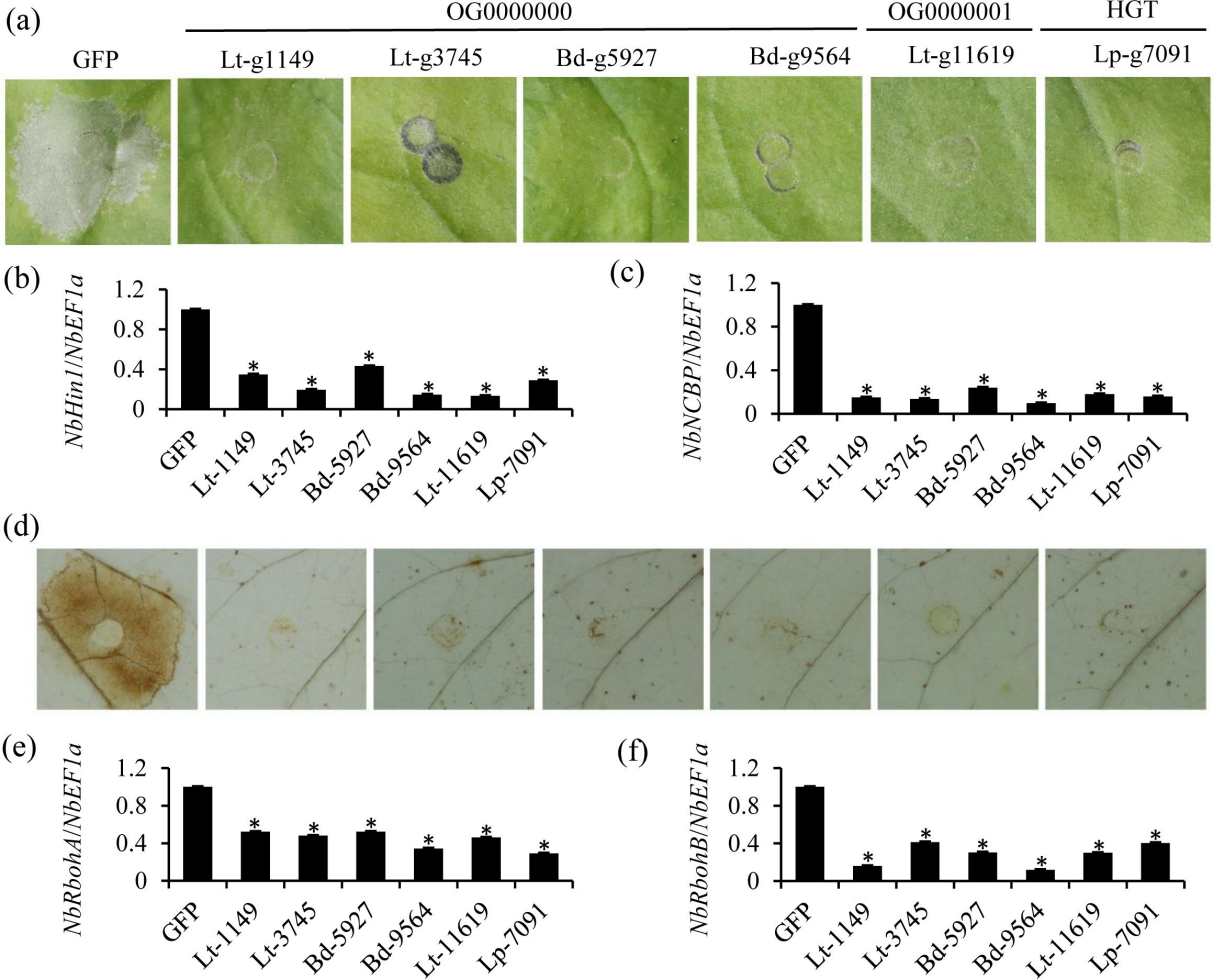
Core and HGT CSEPs inhibit Bax-triggered cell death and ROS signaling in *N. benthamiana*. (**a**) Suppression of BAX-induced cell death by Botryosphaeriaceae CSEPs in *Nicotiana benthamiana*. Leaves of *N. benthamiana* were infiltrated with *Agrobacterium tumefaciens* GV3101 cells carrying either CSEP genes or the GFP gene, followed 12 h later by expression of the mouse *Bax* gene. Representative images were captured 3 days after *Bax* infiltration. (**b and c**) Expression of (**b**) *NbHin1* and (**c**) *NbNCBP* in *N. benthamiana* leaves induced by Bax was significantly inhibited by co-expression with core CSEPs and HGT CSEPs in *N. benthamiana* leaves, as compared with co-expression with GFP. The expression of *NbHin1* and *NbNCBP* in *N. benthamiana* leaves 24 h after BAX infiltration was measured using quantitative real-time RT-PCR (qRT-PCR) assays. The *NbEF1α* gene of *N. benthamiana* was used as an internal control. (**d**) Reactive oxygen species (ROS) induced by BAX was attenuated by co-expression with core CSEPs and HGT CSEPs in *N. benthamiana* leaves, as compared with co-expression with GFP. (e–f) Expression of (**e**) *NbRbohA*, and (**f**) *NbRbohB* in *N. benthamiana* leaves induced by Bax was significantly inhibited by co-expression with core CSEPs and HGT CSEPs in *N. benthamiana* leaves, as compared with co-expression with GFP. The expression of *NbRbohA*, and *NbRbohB* in *N. benthamiana* leaves 24 h after BAX infiltration was measured using qRT-PCR assays. The *NbEF1α* gene of *N. benthamiana* was used as an internal control. *P < 0.05.

The ability to suppress BAX-induced cell death has been proven to be an effective method for identifying immune-suppressive effectors in phytopathogens (31, 77). To investigate whether CSEP genes from the core categories within the Botryosphaeriaceae regulate plant immunity, agrobacteria strains with CSEPs lacking signal peptides were infiltrated into tobacco leaves. Twelve hours later, BAX was infiltrated. The results showed that the control GFP did not inhibit BAX-induced cell necrosis, while in leaves expressing the six CSEPs, the symptoms induced by BAX were suppressed (Fig. 6a). Consistently, compared to leaves expressing only GFP, ion leakage in tobacco leaves expressing the six CSEPs was significantly reduced (Fig. S7). Gene expression analysis using RT-PCR showed that the six CSEPs, BAX, and GFP were all stably expressed (Fig. S8). In addition, expression of cell death marker genes, such as *NbHin1* and *NbNCBP*, was dramatically suppressed in tobacco leaves expressing the six CSEPs compared with the control GFP (Fig. 6b and c). To investigate the ability of CSEPs to suppress cell death symptoms, we evaluated their effect on BAX-induced reactive oxygen species (ROS) production in tobacco leaves. The results showed that expression of CSEPs significantly suppressed BAX-induced ROS accumulation compared to the GFP control (Fig. 6d). Furthermore, the expression of genes encoding NADPH oxidases critical for the ROS burst, such as *NbRbohA* and *NbRbohB*, was significantly down-regulated in the CSEP-expressing leaves compared to the GFP control (Fig. 6e and f).

**Fig 7:**
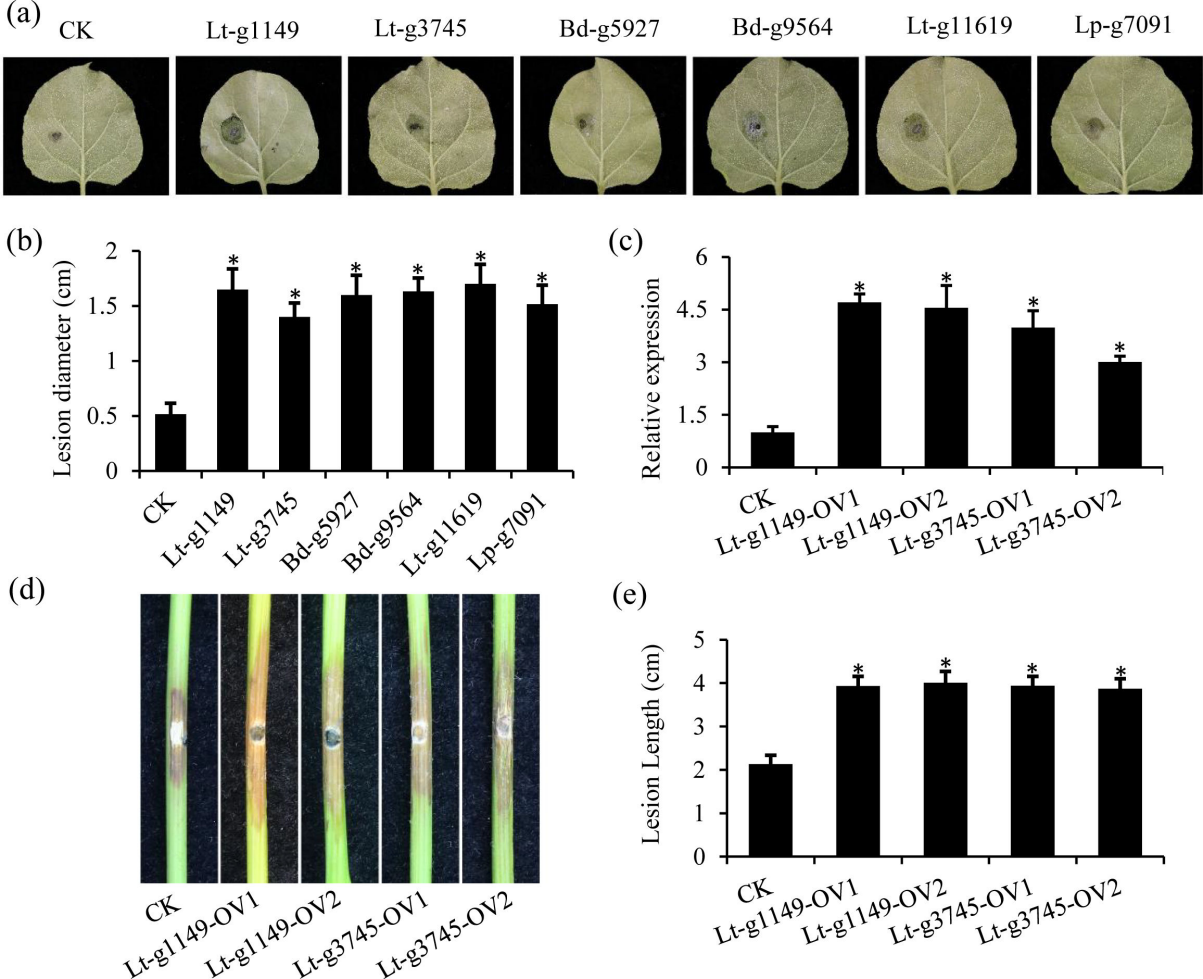
Botryosphaeriaceae CSEPs enhance *L. theobromae* virulence in *Nicotiana benthamiana* and grape. (**a**) Transient overexpression of Botryosphaeriaceae CSEPs in *N. benthamiana* enhances susceptibility to *L. theobromae*. A representative leaf exhibiting disease lesions following inoculation with *L. theobromae* strain CSS-01s is shown, photographed 3–5 days post-inoculation (dpi). (**b**) Average lesion diameters on *N. benthamiana* leaves inoculated with *L. theobromae* CSS-01s (CK), Lt-g1149, Lt-g3745, Bd-g5927, Bd-g9564, Lt-g11619, and Lp-g7091. Error bars indicate ± standard deviation (SD). The infection assays were repeated three times independently. Statistical significance was determined using Student’s *t*-test; *P < 0.05 when comparing each experimental condition to the CK control. (**c**) Disease symptoms on grape shoots were observed about 5 days after inoculation with the *L. theobromae* wild-type and overexpression strains. The wild-type CSS-01s, Lt-g1149-overexpression, and Lt-g3745-overexpression strains were inoculated into detached grape shoots of Summer Black. CK represents the wild-type strain CSS-01s. Scale bar, 1 cm. (**d**) Lesion lengths on grape shoots 5 days after inoculation with the *L. theobromae* wild-type CSS-01s and overexpression strains. (**e**) The relative expression levels of Lt-g1149 and Lt-g3745 expression in *L. theobromae* transformants overexpressing Lt-g1149 (OV1 and OV2) or transformants with overexpressing Lt-g3745 (OV1 and OV2) by using qRT-PCR. The Actin gene of *L. theobromae* was used as an internal control for transcript normalization.

To further explore the biological function of CSEPs, we transiently expressed these six CSEPs in *N. benthamiana* and then inoculated them with *L. theobromae* CSS-01s. The results showed that compared to the control, the lesion diameter of plants expressing the CSEPs was significantly increased (Fig. 7a and b). Subcellular localization assay showed that these CSEPs were properly expressed in *N. benthamiana* (Fig. S9). To assess the contribution of CSEPs to the pathogenicity of *L. theobromae*, we generated stable Lt-g1149 and Lt-g3745-overexpressing transformants via PEG-mediated protoplast transformation. qRT-PCR analysis confirmed that these transformants exhibited significantly higher expression than the wild-type strain (Fig. 7c). Pathogenicity assays on “summer black” grapevine stems revealed that inoculation with the Lt-g1149 and Lt-g3745-overexpressing transformants produced significantly longer lesions compared to inoculation with the wild-type control (Fig. 7d and e). These results suggested that expression of the CSEPs in core categories and a potential HGT gene in the dispensable categories can increase plant susceptibility to *L. theobromae*. Collectively, these findings indicate that these CSEPs play a key virulence role during successful host infection by effectively suppressing critical responses of the plant immune system.

## DISCUSSION

Botryosphaeriaceae species are among the most diverse fungal taxa, widely distributed across various ecosystems. They play a crucial role in agriculture and forestry, exhibiting versatile lifestyles as pathogens, endophytes, parasites, and saprobes. Their complex pathogenic mechanisms as opportunistic pathogens and the absence of effective control methods pose a major challenge for prevention and control. Thus, it is vital to deeply understand their pathogenesis, with effector proteins playing a key role in this process.

Despite advances in computational approaches, the identification of fungal effector repertoires remains inherently limited. Most effector prediction tools are trained on a relatively small number of experimentally validated fungal effectors, while effector proteins themselves display high sequence diversity and limited conservation. Consequently, predicted effector sets are unlikely to be fully comprehensive, and some bona fide effectors may be missed. To assess the robustness of our predictions, we applied an alternative effector prediction tool and compared the resulting candidate secreted effector proteins with those identified by our primary pipeline. The high consistency observed between different tools, with approximately 99% overlap among predicted CSEPs, supports the reliability of our predictions under the current analytical framework. Given the highly variable and rapidly evolving nature of effector proteins, it is likely that many bona fide effectors remain undiscovered. Accordingly, future progress in fungal effector research will rely on the development of more accurate prediction algorithms, the incorporation of larger and more diverse training data sets, and comprehensive experimental validation.

Effectors play key roles in pathogen-plant interaction. One notable characteristic of effector proteins is low conservation, exhibiting significant differences across different species (11). In this study, through super pan-effectome analysis, we identified both conserved and diversified effector gene families in Botryosphaeriaceae. Previous studies on other pathogens discovered that there exist some conserved effector proteins that play crucial roles during the infection process. For example, the conserved effector CSEP0214 from *Blumeria hordei* targets the VPS18 protein, disrupting the endomembrane trafficking pathway (78). The conserved effector NIS1 in *Colletotrichum orbiculare* targets the plant co-receptor BAK1 to regulate disease resistance (79). Pathogenic fungi of the genera *Magnaporthe* and *Colletotrichum* hijack the plant phosphate signaling pathway by secreting conserved Nudix hydrolase effectors to facilitate their own infection (80). The highly conserved RXLR and CRN effector proteins, widely present in oomycetes, can evade, suppress, or manipulate host defense mechanisms through diverse means (81, 82).

Effector protein evolution is a complex and dynamic process (83). In this study, core and soft-core categories, which exhibited greater evolutionary conservation, were likely retained through long-term natural selection and played a pivotal role in the pathogenicity of pathogens. The diversity of R-proteins places effector proteins under rapid evolutionary selection, resulting in a higher evolutionary rate for effector proteins (18). Direct recognition by R-proteins appears to drive point mutations in effector genes, resulting in amino acid substitutions and sequence diversification of effector proteins, whereas indirect recognition seems to promote the loss of effector genes (84). For example, one of the main mechanisms by which Avr9 and Avr4E effector proteins in *Cladosporium fulvum* acquire toxicity is through the loss of effector genes (85, 86). The frequent loss of genes encoding effector proteins observed across all analyzed species suggests that gene loss may represent one of the major evolutionary mechanisms underlying effector diversity in Botryosphaeriaceae.

Additionally, horizontal gene transfer serves as another crucial pathway for effector protein evolution by enabling pathogens to swiftly acquire new virulence factors or adaptive traits, consequently boosting their pathogenicity and competitive survival capabilities (87). For example, *Fusarium oxysporum* can become a pathogen capable of infecting tomatoes through the acquisition of lineage-specific chromosomes via horizontal gene transfer, even if it was originally non-pathogenic (88). Other studies have shown that fungi in Botryosphaeriaceae exhibit horizontal gene transfer during evolution, enabling them to acquire infection-related genes from other fungi (55). In this study, we also identified numerous horizontally transferred effector genes from diverse effector families within the Botryosphaeriaceae. Most of these genes originated from species in the class Sordariomycetes, suggesting that inter-kingdom exchange of effector genes may represent an important mechanism by which Botryosphaeriaceae expand their pathogenicity repertoire. In this study, we found a case where *L. pseudotheobromae* acquired a cutinase effector protein gene from the Sordariomycetes, and experimental evidence demonstrated that this gene can regulate plant immunity. Moreover, research has shown that the cutinase effector protein Pst22751 from *Puccinia striiformis* can inhibit the accumulation of active oxygen in wheat and suppress programmed cell death induced by BAX (89).

We successfully identified three conserved effector gene families, in which the core genes were expressed early after infecting the host. Two out of three conserved effector gene families were annotated as pectin lyase (OG0000000) and peptidase inhibitor family (OG0000001), respectively. They are likely to play a crucial role in the infection process of pathogens, such as inhibiting the host defense mechanisms and interfering with the host normal physiological functions (90, 91). The pectin lyase family underwent gene family expansion, including an ancient event that occurred before the divergence of Botryosphaeriaceae into genera. The ancient expansion of pectin lyase genes in Botryosphaeriaceae may have facilitated the transition of their ancestor from a saprotrophic to a pathogenic lifestyle (55). Expansions of pectin-decomposition genes were also identified in woody-inhabiting *Valsa* canker pathogens (90). Therefore, the expansion of genes involved in pectin degradation may represent an important feature contributing to the infection strategies of woody plant pathogens. Our functional validation results also indicated that these conserved effector proteins are critical for the pathogenicity of the pathogen. Therefore, gene editing technologies can be used to knock out or silence these conserved genes, to conduct deeper research on their pathogenic mechanisms. These conserved genes can also be utilized as new targets for developing novel broad-spectrum fungicides against branch diseases caused by Botryosphaeriaceae fungi, providing strong support and new strategies for disease control. Additionally, there is one conserved effector gene family (OG0000002) whose function remains unclear. In future studies, further exploration of the function of this gene family could provide a more comprehensive understanding of the pathogenic mechanisms of Botryosphaeriaceae fungi.

As research on effector proteins continues to deepen, their significant value in agricultural production and disease control has become increasingly apparent. The future of studying effector proteins in pathogenic fungi holds immense potential, with numerous directions waiting to be explored. By utilizing pan-effectome technology, the scope of research can be expanded to include systematic analysis of a broader range of fungal strains across various pathogenic species. This approach could uncover additional conserved and non-conserved effector proteins, continually enriching our understanding of the pathogenic mechanisms of pathogenic fungi as a whole.

## Data Availability

The publicly available RNA-Seq data used in this study were obtained from the Gene Expression Omnibus database under accession number GSE190625.

## SUPPLEMENTAL MATERIAL

Supplemental figures aem.01619-25-s0001.docx Fig. S1 to S9.

Fig. S10 aem.01619-25-s0002.pdf Full evolutionary tree of laps.g7091.t1.

Supplemental tables aem.01619-25-s0003.xlsx Tables S1 to S5.

ASM does not own the copyrights to Supplemental Material that may be linked to, or accessed through, an article. The authors have granted ASM a non-exclusive, world-wide license to publish the Supplemental Material files. Please contact the corresponding author directly for reuse.
