# Supplementary material for: Comprehensive pan-effectome investigation reveals central effector genes in woody plant pathogen Botryosphaeriaceae
Source: Appl Environ Microbiol. 2026 Apr 6;92(5):e01619-25. doi: 10.1128/aem.01619-25 (PMC13188853; doi:10.1128/aem.01619-25)
Supplement: Supplemental figures — Fig. S1 to S9. [file aem.01619-25-s0001.docx]

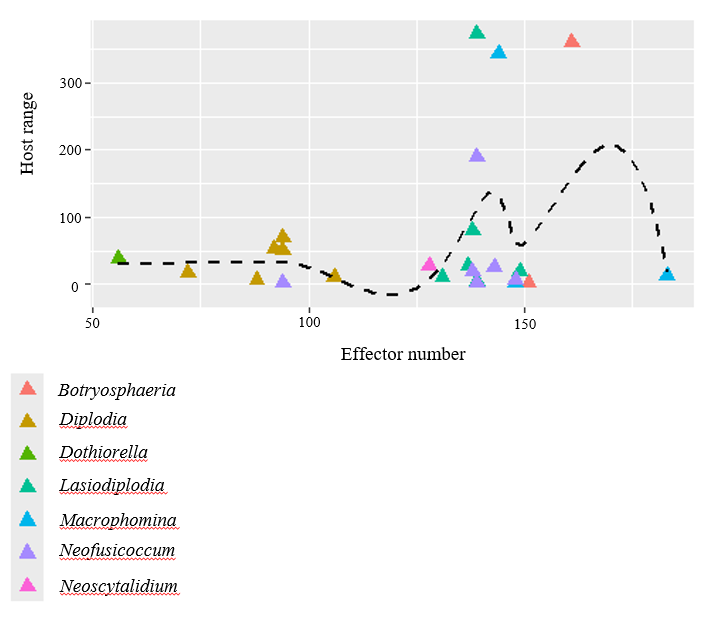


**Fig. S1 Relationship between the number of Candidate Secreted Effector Proteins (CSEPs) and host range in fungal species.** Host range data were obtained from the USDA Fungal Databases (https://fungi.ars.usda.gov/).


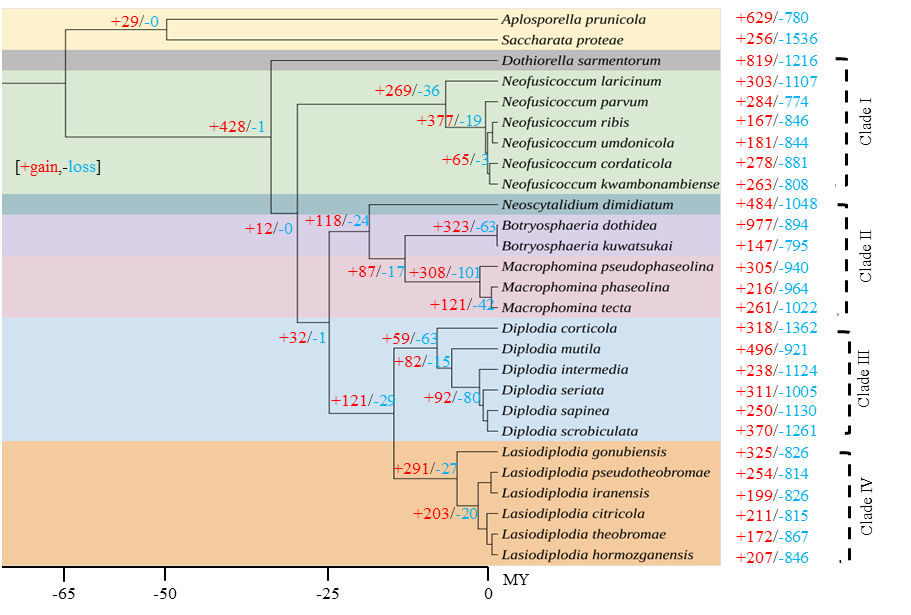


**Fig. S2 Genome-wide analysis of gene gain or loss in Botryosphaeriaceae**.


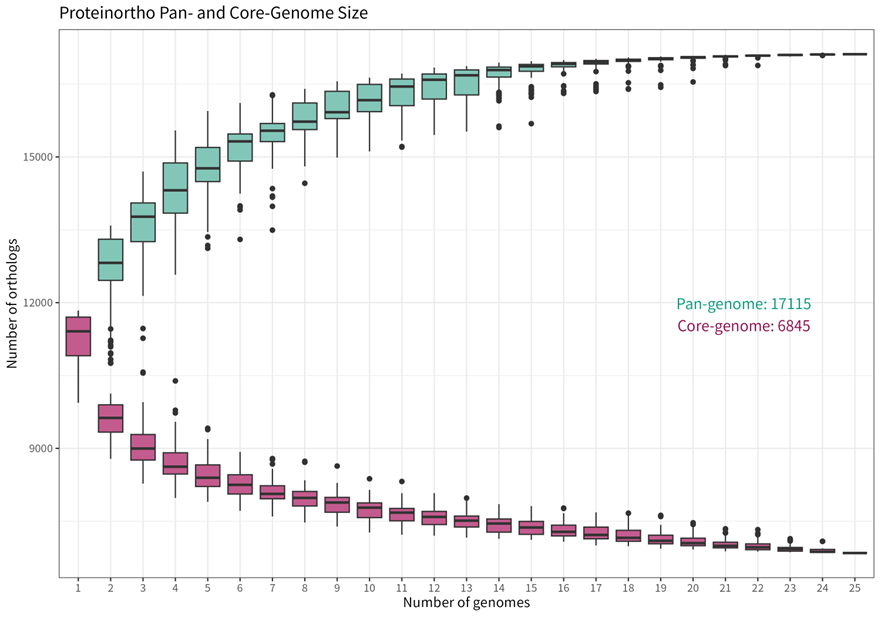


**Fig. S3 Changes in the number of pan-proteome and core-proteome as the genome numbers increase.**


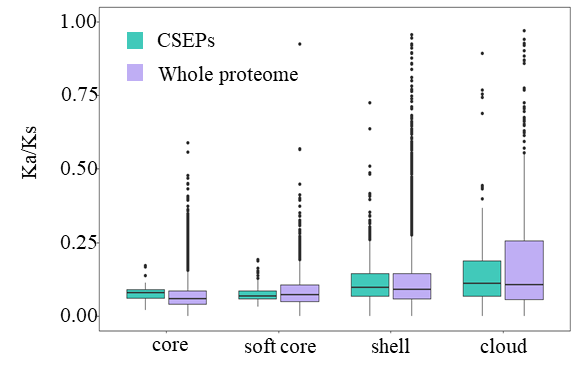


**Fig. S4 Comparative analysis of selection pressure of CSEPs and proteins from different categories.**


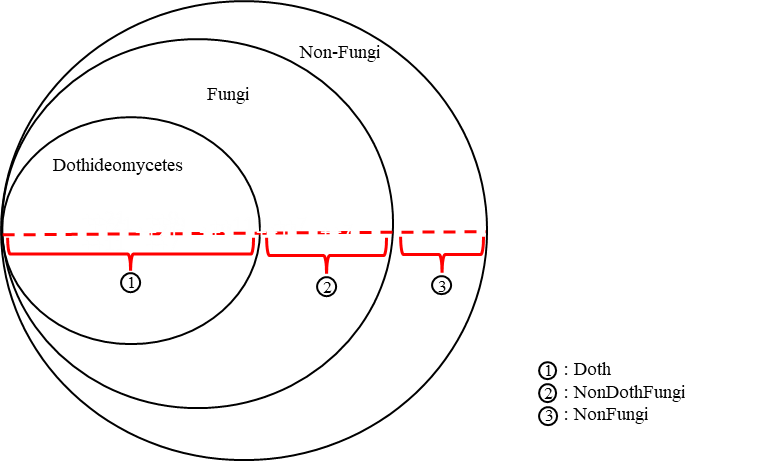


**Fig. S5 Schematic diagram.** classification of Botryosphaeriaceae genes into different evolution groups base on the phylogenetic distance of the species from its best-matched non-Botryosphaeriaceae blast hit in NR database. ➀: the species of top hits is within Dothideomycetes. ➁: the species of top hits is within non-Dothideomycetes but within Fungi. ➂: the species of top hits if from non-Fungi organisms.


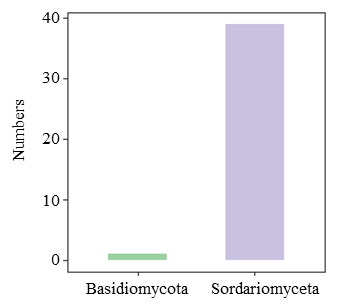


**Fig. S6 The donor groups of horizontally transferred genes.**


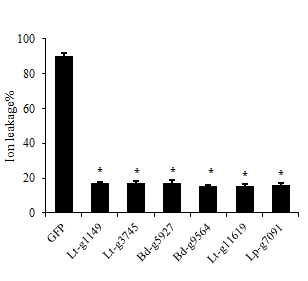


**Fig. S7 Quantification of cell death by electrolyte leakage assay. Data represent means ± standard error (SE), based on six independent replicates.**


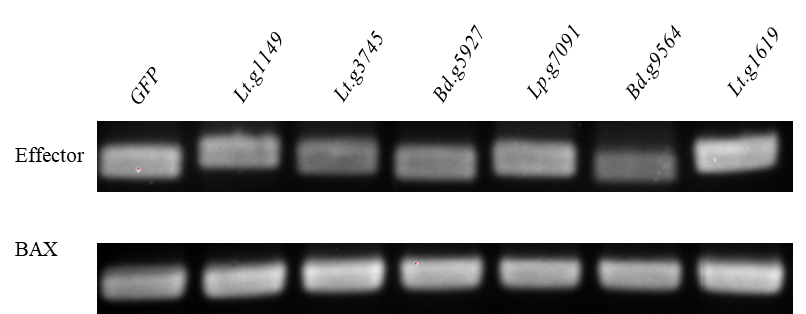


**Fig. S8 RT-PCR analysis of the expression of Botryosphaeriaceae effectors in agroinfiltrated *N. benthamiana* leaves.** Total RNAs was extracted from *N. benthamiana* leaves at 24 hours post-agroinfiltration. The *Actin* gene of *N. benthamiana* was used as an internal reference control for normalization.


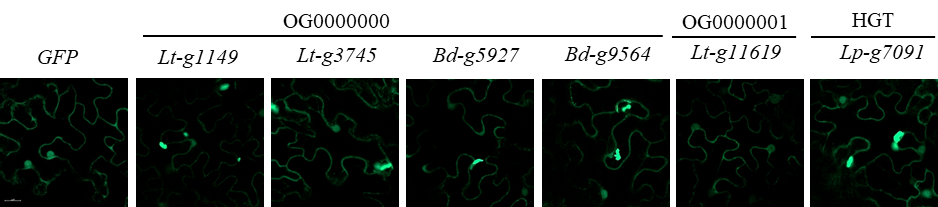


**Fig. S9** **Subcellular localization of Botryosphaeriaceae effectors in *N. benthamiana* epidermal cells**. The subcellular localization of core Botryosphaeriaceae effectors was examined by transient expression in *N. benthamiana* epidermal cells. Fluorescence signals of effector proteins, tagged with fluorescent markers, or the GFP control were observed using a confocal laser scanning microscope (CLSM) 48 hours post agroinfiltration. Scale bars = 20 μm.
