# Supplementary figures and images for: Comprehensive pan-effectome investigation reveals central effector genes in woody plant pathogen Botryosphaeriaceae
Source: Appl Environ Microbiol. 2026 Apr 6;92(5):e01619-25. doi: 10.1128/aem.01619-25 (PMC13188853; doi:10.1128/aem.01619-25)

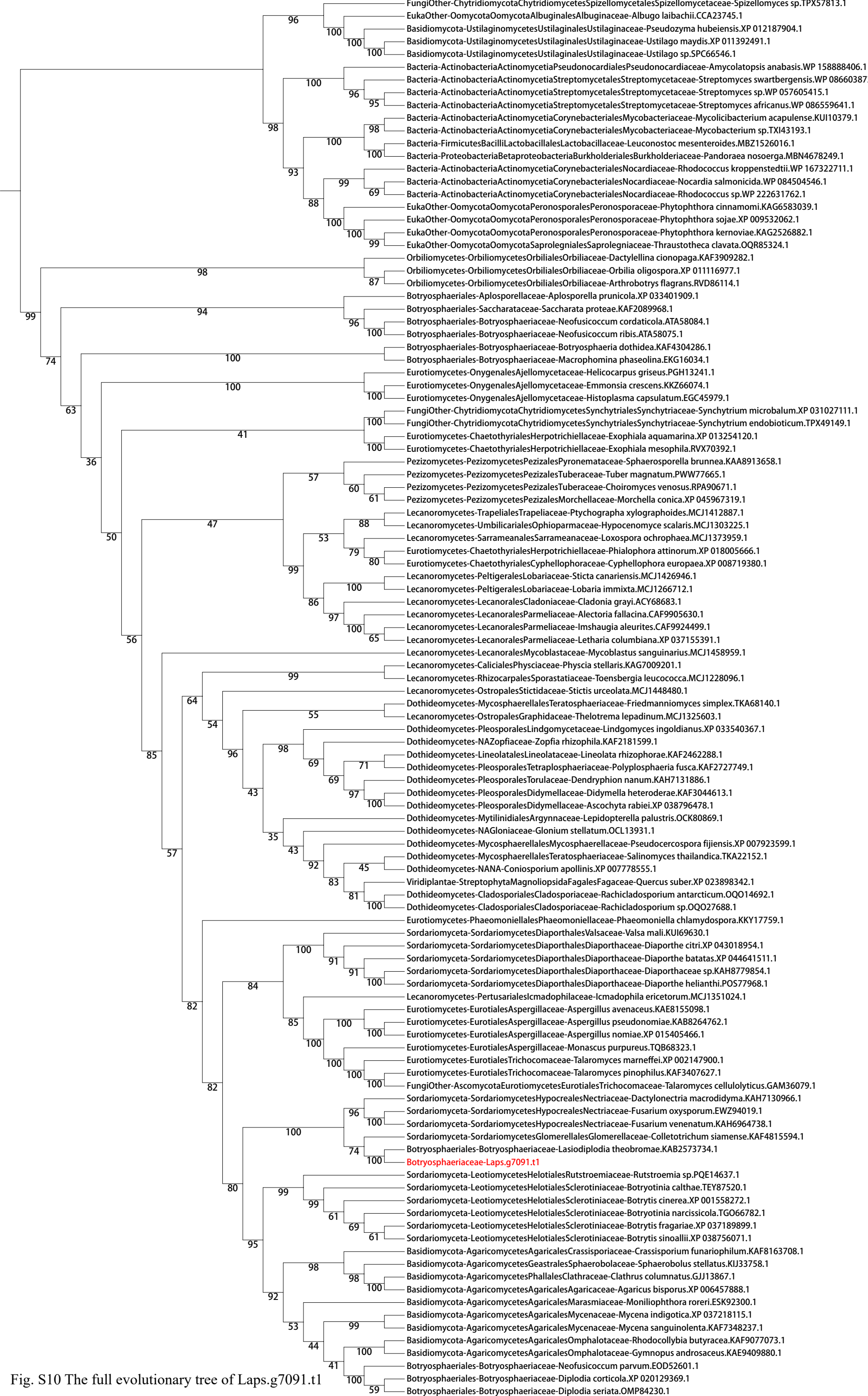

Supplement: Fig. S10 — Full evolutionary tree of laps.g7091.t1. [file aem.01619-25-s0002.pdf]
